# Mutational landscape of radiation-associated angiosarcoma of the breast

**DOI:** 10.18632/oncotarget.24273

**Published:** 2018-01-19

**Authors:** Bryan J. Thibodeau, Vincent Lavergne, Nayana Dekhne, Pamela Benitez, Mitual Amin, Samreen Ahmed, Jean L. Nakamura, Philip R. Davidson, Alice O. Nakamura, Inga S. Grills, Peter Y. Chen, Jessica Wobb, George D. Wilson

**Affiliations:** ^1^ Beaumont BioBank, Beaumont Health, Royal Oak, MI, USA; ^2^ Department of Radiation Oncology, University of California, San Francisco, CA, USA; ^3^ Department of Surgery, Beaumont Health, Royal Oak, MI, USA; ^4^ Department of Pathology, Beaumont Health, Royal Oak, MI, USA; ^5^ Department of Finance and Statistical Analysis, University of Alberta, Edmonton, Alberta, Canada; ^6^ Department of Radiation Oncology, Beaumont Health, Royal Oak, MI, USA

**Keywords:** angiosarcoma, radiation-associated, breast, mutational signature, next generation sequencing

## Abstract

**Purpose:**

Radiation-associated breast angiosarcomas are a rare complication of radiation therapy for breast carcinoma. With relatively little is known about the genetic abnormalities present in these secondary tumors, we examined genomic variation in biospecimens from radiation-associated breast angiosarcomas.

**Experimental Design:**

Patients were identified that had a previous breast cancer diagnosis, received radiation therapy, and developed angiosarcoma in the ipsilateral breast as the earlier cancer. Tumor regions were isolated from archival blocks using subsequent laser capture microdissection. Next generation sequencing was performed using a targeted panel of 160 cancer-related genes. Genomic variants were identified for mutation and trinucleotide-based mutational signature analysis.

**Results:**

44 variants in 34 genes were found in more than two thirds of the cases; this included 12 variants identified as potentially deleterious. Of particular note, the BRCA1 DNA damage response pathway was highly enriched with genetic variation. In a comparison to local recurrences, 14 variants in 11 genes were present in both the primary and recurrent lesions including variants in genes associated with the DNA damage response machinery. Furthermore, the mutational signature analysis shows that a previously defined IR signature is present in almost all of the current samples characterized by predominantly C→T substitutions.

**Conclusions:**

While radiation-associated breast angiosarcomas are relatively uncommon, their prognosis is very poor. These data demonstrate a mutational pattern associated with genes involved in DNA repair. While important in revealing the biology behind these tumors, it may also suggest new treatment strategies that will prove successful.

## INTRODUCTION

Angiosarcomas are a relatively rare histological subtype of sarcomas and represent approximately 1% of all sarcomas [1]. They display remarkable clinical heterogeneity and can occur anywhere in the body, although the breast is a frequent location. Secondary sarcomas are a recognized complication of radiation therapy for breast carcinoma and are associated with poor prognosis [2]. The estimated incidence of treatment-associated breast angiosarcoma is 0.002–0.05% per year corresponding to ∼40% of all radiation-associated sarcomas that develop after radiotherapy [3]. Radiation-associated breast angiosarcomas have poor prognosis and relatively little is known about the genetic abnormalities present in these secondary tumors. Recent evidence has implicated amplification of the 8q24.2 region containing the *MYC* gene in secondary but not in primary breast angiosarcomas [4]. High expression of the Myc protein was found to be associated with amplification, suggesting that MYC amplification may be implicated in the pathology of secondary breast angiosarcomas [5].

A recent study of 7 primary breast angiosarcomas and 18 secondary breast angiosarcomas arising in the irradiation field of a radiotherapy were analyzed for copy number alterations and differential gene expression using Affymetrix SNP 6.0 Array and Affymetrix Exon Arrays [6]. This study showed that two transcriptome signatures of the radiation tumorigenesis coexisted in these tumors. One was histology specific and correctly discriminated 100% of the primary tumors from the radiation-associated tumors. The deregulation of marker genes, including podoplanin (PDPN), prospero homeobox 1 (PROX-1), vascular endothelial growth factor 3 (VEGFR3) and endothelin receptor A (EDNRA), suggests that the radiation-associated breast angiosarcomas developed from radiation-stimulated lymphatic endothelial cells. In addition, the authors showed that the high rate of MYC amplification found in radiation-associated breast angiosarcomas is likely a consequence of genome instability initiated by ionizing radiation; however, they suggest that it is not a marker of the radiation tumorigenesis since it was also observed at a low rate in primary tumors.

There is currently no data on specific mutations in radiation-associated breast angiosarcomas. In this study we used next generation DNA sequencing to investigate genomic variation specific to radiation-associated angiosarcomas. To assess the similarity of our radiation-associated breast angiosarcomas to other radiation-induced malignancies described in the literature [7–9], we performed non-negative matrix factorization (NMF) trinucleotide mutational signature analysis. In addition we used this variation to determine if particular signaling pathways were preferentially altered.

## RESULTS

### Patient data

Table 1 includes clinical and demographic data for 13 cases of radiation-associated angiosarcoma and 3 cases of sporadic angiosarcoma. Clinical data for the angiosarcoma as well as the initial breast cancer is included for patients with radiation-associated angiosarcoma. All but one of the radiation-associated angiosarcomas were treated by surgical mastectomy, and 9 of the 13 had grade 3 disease. While there were 4 local recurrences, only 1 went on to develop distant metastasis. Interestingly the earlier breast cancer tended to be stage I or II and estrogen receptor positive. The vast majority of the initial breast cancers were treated with whole breast irradiation with more than 60% also receiving hormone therapy. The median interval between the initial breast cancer and the radiation-associated angiosarcoma was 6.8 years with a range from 3.9 to 13.5 years.

**Table 1:** Patient data

Patient ID	Angio Type	age dx	Angio Surgery type	grade	chemo	LR	DM	Interval (month)	Earlier Breast Cancer				
									Stage	ER/PR/Her2	chemo	hormones	XRT
AX5612	Rad-Ind	74	simple mastectomy	3	no	+	–	109	IA	+ / ND / ND	no	novaldex	WBI
AX5613	Rad-Ind	77	simple mastectomy	3	no	+	–	87	IA	+ / ND / –	no	tamoxifen	WBI
AX5617	Rad-Ind	86	incisional biopsies only	3	no (refused)	–	–	68	IA	98% / 2% / 0+	no	no	WBI
AX5618	Rad-Ind	59	mastectomy	2	doxycycline, ifosfamide, mesnax4	+	+	162	IA	94% / ND / ND	no	tamoxifen	WBI
AX5615	Rad-Ind	74	simple mastectomy	3	no	–	–	81	IIA	1% / 2% / ND	no	no	WBI
AX5626	Rad-Ind	75	simple mastectomy	3	no	–	–	105	IA	– / – / ND	no	tamoxifen	WBI
AX5625	Rad-Ind	62	simple mastectomy	3	yes (unknown)	–	–	75	IA	99%/12%/0+	no	arimidex	WBI
AX5622	Rad-Ind	^**^	mastectomy	^**^	^**^	^**^	^**^	^**^	^**^	^**^	^**^	raloxifene	^**^
AX5628	Rad-Ind	82	simple mastectomy	3	no	–	–	107	IIA	74%/ND / –	no	no	WBI
AX5616	Rad-Ind	82	mastectomy/wide local excision with graft closure	3	no	+	–	60	IA	91% / 16% / 0+	no	no	3D-CRT
AX5619	Rad-Ind	44	simple mastectomy	2	neoadjuvant taxol	–	–	75	IIA	67%/73% / –	ACT	tamoxifen	WBI
AX5614	Rad-Ind	^**^	simple mastectomy	2	neoadjuvant taxol	–	–	^**^	^**^	^**^	^**^	^**^	^**^
AX5621	Rad-Ind	88	nodule excision	3	TMZ	–	–	47	IIIA	100%/ 87% / 0+	no	tamoxifen	WBI
AX5609	Sporadic	39	^**^	^**^	no	^**^	^**^						
AX5627	Sporadic	75	partial resection		no	–	–						
AX5630	Sporadic	70	simple mastectomy	3	^**^	–	+						

Angiosarcoma type: radiation-induced (Rad-Ind) and sporadic Local recurrence (LR): yes (+), no (–). Distant metastasis (DM): yes (+), no (–). Estrogen Receptor (ER)/Progesterone Receptor (PR)/Her2: non-determined (ND). Radiation treatment (XRT): whole breast (WBI), 3D-CRT (3-dimensional conformal radiotherapy). ^**^: not available/unknown.

### Radiation-associated angiosarcoma

Next generation sequencing was performed on the Illumina NextSeq using a panel of 160 cancer-related genes. Variants were initially filtered to include only those found in more than two thirds (≥ 9 of 13) of the cases of radiation-associated angiosarcoma. There were 44 variants in 34 different genes (Supplementary Table 2) including 12 variants that were identified as potentially deleterious (Table 2). Variants were recognized as potentially deleterious by meeting one of the following criteria: (i) categorized as pathogenic or likely pathogenic based upon ACMG guidelines, (ii) listed in HGMD/ClinVar, or (iii) having a CADD (Combined Annotation Dependent Depletion) score greater than 20. Among these variants, 3 missense mutations (EGFR c.1496G > A / p.Cys499Tyr; BRAF c.1915G > A / and c.1894C > T / p.Pro632Ser) were found in all 13 cases. The p.Val639Ile mutation in BRAF has been annotated in COSMIC as associated with squamous-cell carcinoma in the lung.

**Table 2:** Variants found in more than two-thirds (≥ 9 of 13) of the radiation-induced angiosarcoma cases and are potentially deleterious (pathogenic or likely pathogenic by ACMG guidelines, listed in HGMD/ClinVar, or predicted to be deleterious by having a CADD score > 20)

Chr	Position	Gene Symbol	Ref. Allele	Alt. Allele	Protein Variant	Cases With Variant	Impact	Classification
1	27,100,182	ARID1A	GC	–	p.Q1327fs^*^10	9	FS	Likely Pathogenic
1	193,111,246	CDC73	AG	–	–	11	NC	VUS
2	47,635,536	MSH2	T	–	–	10	NC	VUS
2	48,032,881	MSH6	ATCT	–	–	11	NC	VUS
5	56,180,645	MAP3K1	G	T	p.W1325L	12	MS	VUS
5	170,827,869	NPM1	T	A	p.N203K	12	MS	VUS
7	55,228,029	EGFR	G	A	p.C499Y	13	MS	VUS
7	140,449,164	BRAF	C	T	p.V639I	13	MS	VUS
7	140,449,185	BRAF	G	A	p.P632S	13	MS	VUS
13	32,907,546	BRCA2	T	–	–	12	NC	VUS
15	40,501,853	BUB1B	C	T	p.P721S	11	MS	VUS
17	29,545,994	NF1	–	T	–	12	NC	VUS

Impact: frameshift (FS), missense (MS), non-coding (NC). ACMG Classification: unknown significance (VUS).

**Figure 1:**
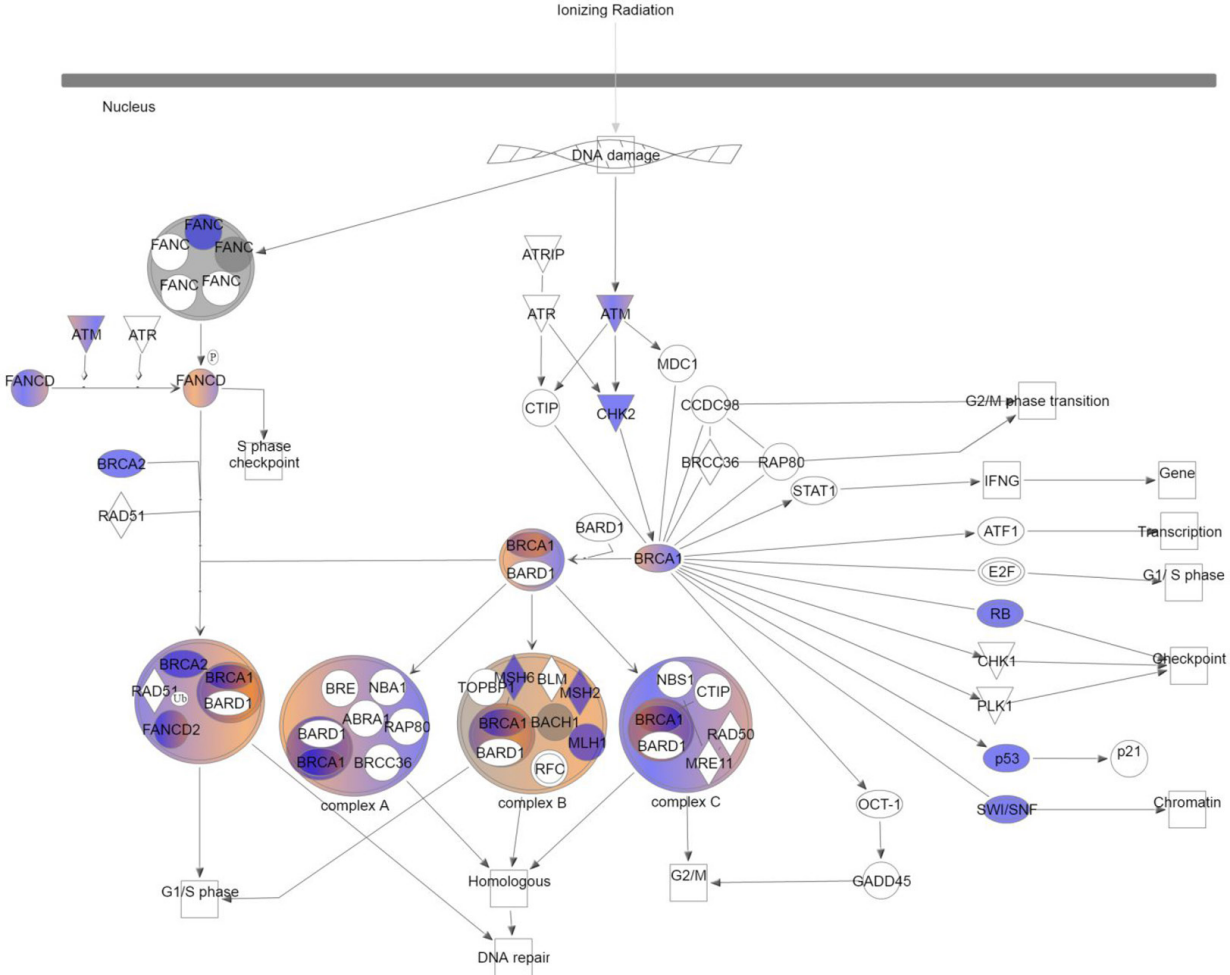
Role of BRCA1 in DNA damage response Color indicates variants were present in at least 1 case of radiation-induced angiosarcoma after filtering common variants. Blue: loss of function; red: gain of function; grey: inferred normal.

Further characterization of the variants present in the radiation-associated angiosarcomas was done with Ingenuity Variant Analysis to investigate signaling pathways preferentially affected in radiation-associated angiosarcomas. The “role of BRCA1 in DNA damage response” was highly enriched with genetic variation. In order to focus on variants shown to have a damaging effect, filtering was applied to include only those identified as: (i) pathogenic or likely pathogenic by HCMG, (ii) damaging or activating by SIFT function prediction [10], or (iii) probably damaging, possible damaging, or damaging by PolyPhen2 function prediction [11]. This demonstrated that all 13 cases had at least 2 genes affected in this pathway (Figure 1). In total, 17 different genes in the DNA damage response system contained a variant in one of the cases of radiation-associated angiosarcoma (Supplementary Table 3). Each patient sample had an average of 6 genes with a non-synonymous variant (range 2–11).

### Comparison to local recurrence

In addition to the 13 radiation-associated angiosarcomas, two patients had tissue available from the resulting local recurrence (patient ID AX5613 and AX5616 in Table 1). Only 2 variants (the missense variant BUB1B c.2161C > A / p.Pro721Thr and the synonymous variant EP300 c.2871T > A / p.T957T) occurred in the primary angiosarcoma that did not occur in the local recurrence; however, 14 variants in 11 genes were present in both the primary and recurrent lesions (including 2 missense variants and 1 splice site loss, Table 3). This includes variants in 4 genes (ATM, BRCA1, BRCA2, MSH6) associated with the DNA damage response machinery. Also of interest may be the 14 variants (including 7 non-synonymous variants) in 11 genes that are present in the local recurrence and not in the primary angiosarcoma (Supplementary Table 4). Among these are 6 variants that are predicted to be pathogenic / damaging by the IVA software, including variants in JAK1 (c.3047T > A, / p.Val1016Glu), TERT (c.1883A > C / p.Asp628Ala), KMT2D (c.1185G > T / p.Gln395His), ERCC5 (c.2431A > G / p.Ser1265Gly), DICER1 (c.3572T > A / p.Leu1191*), and EP300 (c.6881T > A / p.Leu2294Gln).

**Table 3:** Variants found in both the primary radiation-induced angiosarcoma as well as the matched local recurrence (*n* = 2 patients)

Chr	Position	Gene Symbol	Ref. Allele	Alt. Allele	Protein Variant	Impact	Classification
1	11,293,378	MTOR	A	–	–	NC	VUS
1	120,510,722	NOTCH2	T	C	p–E414E	syn	Likely Benign
2	48,032,881	MSH6	ATCT	–	–	NC	VUS
5	170,818,300	NPM1	T	–	–	NC	VUS
5	170,827,869	NPM1	T	A	p–N203K	MS	VUS
11	108,188,279	ATM	T	–	–	NC	VUS
11	108,196,725	ATM	AAT	–	–	NC	VUS
13	32,907,546	BRCA2	T	–	–	NC	VUS
15	40,501,853	BUB1B	C	T	p–P721S	MS	VUS
16	2,138,213	TSC2	T	A	–	SSL	VUS
17	41,249,370	BRCA1	A	–	–	NC	VUS
19	4,110,576	MAP2K2	C	A	p–S127S	syn	VUS
22	41,527,414	EP300	T	G	p–V435V	syn	Likely Benign
22	41,565,478	EP300	T	–	–	NC	VUS

Impact: missense (MS), synonymous (syn), splice site loss (SSL), non-coding (NC). ACMG Classification: unknown significance (VUS).

### Specific to sporadic angiosarcoma

In order to better identify variants that may be specific to radiation-associated angiosarcoma, 3 cases of angiosarcoma that developed in the absence of a previous cancer were also examined (Table 1). After variants present in any of the sporadic angiosarcomas were excluded, only 8 variants present in more than half of the radiation-associated angiosarcomas remained (Table 4). However, 2 of these variants, a frame-shift deletion in ARID1A (c.3978_3979delGC / p.Gln1327fs*10) and a missense variant in FANCA (c.1127A > T / p.Gln376Leu), were associated with the “role of BRCA1 in DNA damage response” pathway. Furthermore, there were 9 variants that were present in all 16 patient samples (13 radiation-associated angiosarcomas plus 3 sporadic angiosarcomas). Remarkably none of these were associated with the BRCA1 signaling pathway.

**Table 4:** Variants found in more than half of the radiation-induced angiosarcoma cases and 0 of 3 sporadic angiosarcomas

Chr	Position	Gene Symbol	Ref. Allele	Alt. Allele	Protein Variant	Cases With Variant	Impact	Classification
1	27,100,182	ARID1A	GC	–	p.Q1327fs^*^10	9	FS	Likely Pathogenic
2	47,600,591	EPCAM	T	–	–	9	NC	VUS
7	6,026,541	PMS2	C	T	p.D513N	7	MS	VUS
11	119,156,068	CBL	T	A	p.L578Q	7	MS	VUS
14	23,607,210	SLC7A8	T	A	p.G312G	7	syn	VUS
16	89,858,433	FANCA	T	A	p.Q376L	7	MS	VUS
17	37,657,470	CDK12	T	–	–	7	NC	VUS
22	41,547,890	EP300	T	A	p.T957T	9	syn	VUS

Impact: frameshift (FS), missense (MS), synonymous (syn), non-coding (NC). ACMG Classification: unknown significance (VUS).

### Mutational signature analyses

**Figure 2:**
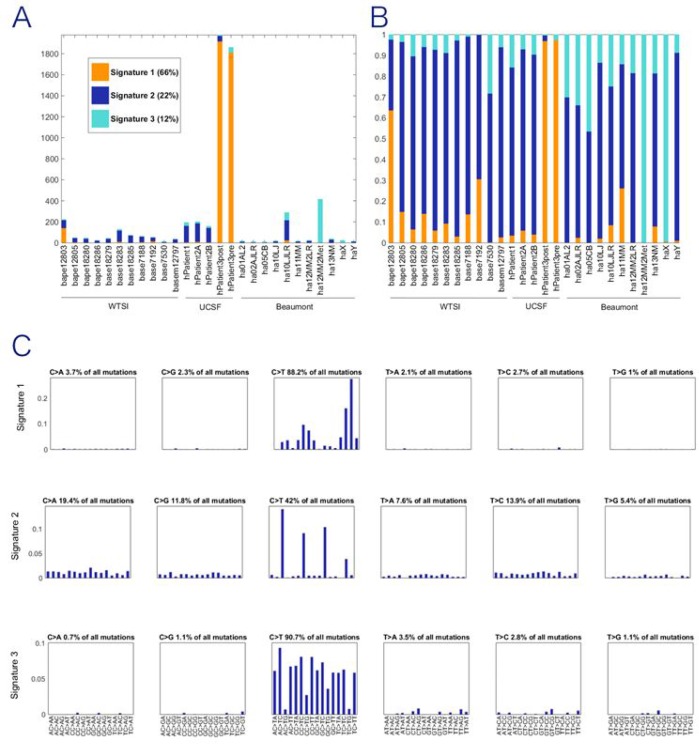
Exposures for NMF trinucleotide mutational signatures for combined dataset WTSI represents the angiosarcomas reported in Behjati et al. (7); UCSF represents the irradiated samples reported in Davidson et al. (9); Beaumont represents the new data first reported in this paper. (**A**) Count of mutations in each tissue sample attributed to each signature. (**B**) Proportion of mutations in each tissue sample attributed to each signature. (**C**) Three discrete mutational signatures were identified the pooled analysis. The plots show the distribution of the six mutation types defined by the pyrimidine base in each signature, as inferred from the NMF procedure. Each sub-graph within a signature represents one substitution (e.g., C**→**A when C in the reference genome is mutated to A in the sample). The bars within each sub-graph include the nucleotides in the reference genome on either side of the mutation location (e.g., AC > AG represents A at 5', C in the reference mutated to A, and G at 3'), 96 substitution types shown. All three signatures are characterized by predominantly C**→**T substitutions but the weights differ markedly by neighboring nucleotides. Signature 1 is defined by the skin cancer exomes and is the recovered UV signature.

In the NMF portion of the study, the goal was to identify a mutational signature consisting of a trinucleotide pattern where variants occur rather than identify specific genes with variation. We utilize two human angiosarcoma datasets: (1) the samples in the current Beaumont breast cancer angiosarcoma dataset with 10 or more mutations and (2) the previously published WTSI dataset with mutation data for 11 angiosarcomas that developed in various anatomical locations [7]. These two datasets, with the data normalized according to equal trinucleotide frequencies, are referred to hereafter as the Bang dataset (B for Beaumont and ang for angiosarcoma) and the WTSIang dataset, respectively. Two human datasets from UCSF were also included. One contained three samples from two patients who developed IR-induced sarcomas (the P12 dataset). The other is for two samples from a UV-associated squamous cell carcinoma of the scalp (the P3 dataset). The trinucleotide mutational signature for UV was previously defined; thus, inclusion of the P3 data serves as a positive control for a non-IR-associated malignancy. The combined P12 and P3 datasets are referred to as the P123 dataset (denoted UCSF in Figure 2A/2B).

Pooling of these datasets results in three stably extracted trinucleotide signatures (Supplementary Figure 1; Figure 2). The 3 signatures extracted from the WTSIang + P123 + Bang dataset are shown in Figure 2C, for which the exposure graph is shown in Figure 2A and 2B. These signatures are not highly correlated with each other (Supplementary Table 5, rows 11–13, cols. I-K), suggesting that they may represent separate mutational processes. One of the three NMF signatures from the full dataset is highly correlated (0.999) with the previously defined UV signature [9], which reflects the inclusion of the P3 data in the full dataset and simply demonstrates that this previously defined signature can still be extracted with the other datasets included along with P3 in the present analysis. Of greater substantive interest, the second of the three signatures extracted from the full dataset has a correlation of 0.790 with the previously defined IRa signature present in known IR-induced human cancers [9, 12]. We see moreover from row 15, col. E of Supplementary Table 5 that even when only the Bang dataset is utilized, the second of the three signatures that can be extracted (albeit with poorer stability properties than for the three signatures extracted from the full dataset) is also correlated 0.706 with the previously defined IRa signature. Thus, whereas also including the WTSIang and the P1–2 and P3 datasets improves the stability of the extracted NMF signatures, the noted correlation with the previously defined IRa signature does not depend on that augmentation of the dataset utilized for NMF.

Figure 2A shows the NMF exposures for the extraction of three signatures from the full dataset. It is clear from this figure that there are far less mutations in the Bang than in the other datasets, and that the P3 skin cancer samples have by far the most mutations. One important property to note for the form of NMF being utilized is that the signatures extracted are not directly affected by even very large differences among the samples in the number of mutations per sample because the input frequencies are converted to relative frequencies as the first step in the NMF iterative process [13].

In Figure 2B, the exposure results have been converted to a proportional representation for each of the signatures. An observation made by Davidson et al. [9] that applies here as well is that all of the extracted signatures are present in almost all of the cancer samples, but the proportionate representations differ greatly for the UV-associated (Signature 1) and the IR-associated samples. Most importantly in terms of questions of interest involving angiosarcomas, the dark blue representing the signature that is highly correlated with the previously defined IRa signature is present in almost all of the Bang samples in similar proportions to its representation in the WTSIang and also the P1–2 dataset samples. Figure 2C displays each of the three signatures. All three signatures are characterized by predominantly C→T substitutions but the weights differ markedly by neighboring nucleotides.

## DISCUSSION

Investigation of radiation-associated angiosarcomas has always proven difficult due to the extremely low incidence [14]. This means that case studies are the predominant type of investigation found in the literature. Despite the low number of patients in each study, there has been significant agreement among them. The interval between primary breast carcinoma and eventual radiation-associated angiosarcoma generally has a median of approximately 7 years with a range between 3 and 25 years [14–19]. This corresponds well with our current cohort that ranged between 3.9 and 13.5 years with a mean of 6.8 years. Additionally disease-free survival was found to be 35% [14] at 2 years with a median of 16 months [18]. Studies also found high levels of recurrence [17] with local recurrence in up to two thirds of patients and metastasis in more than one thirds [20]. Our cohort did not exhibit this high level of recurrence with only 4 of the 13 cases having recurrences. These poor outcomes are a direct result of the difficulty in diagnosing these angiosarcomas following treatment of the primary breast carcinoma [21]. Additionally, while no study currently demonstrates it, some hypothesize that the incidence of radiation-associated angiosarcoma may increase due to the higher usage of radiation in breast conserving techniques in the treatment of breast cancer.

The current study aimed to discover somatic genomic variants common in radiation-induced angiosarcoma; moreover, given the limited data available, signaling pathways were investigated for high levels of variation which may prove important to guide targeted treatments. In the current study we examined a panel of 160 cancer-related genes. In the radiation-associated angiosarcomas, 44 variants were present in at least two thirds of the 13 cases. In order to identify variants that were more likely to be deleterious, these were further filtered based upon ACMG, HGMD, ClinVar, or CADD. This identified 12 variants, including 6 missense variants, 5 small deletions, and 1 single base insertion. Of interest was the missense variant in EGFR that resulted in an amino acid change from cysteine to tyrosine. It was found in all 13 cases of radiation-associated angiosarcoma. This variant is affects the extracellular ligand-binding domain of EGFR and is predicted to affect the function of the protein by both SIFT and PolyPhen-2 function prediction algorithms. The importance of EGFR was suggested in a study of a cell line derived from a radiation-associated angiosarcoma where inhibition of the VEGFR2/EGFR/RET axis resulted in decreased cell proliferation [22]. The only other gene with variants in all 13 cases was BRAF, which had 2 separate variants (chr7:140,449,164 and chr7:140,449,185) present in all cases. Murali et al. also found variation in BRAF including single nucleotide variants and amplification in a study of primary angiosarcomas [23].

In order to identify signaling that is highly altered in the radiation-induced angiosarcomas, we focused on variants that were shown to have a damaging effect on gene function without requiring two thirds of the cases to have a given variant. This identified genes involved in the DNA damage response to be highly altered in the all cases of radiation-associated angiosarcoma. Each case had an average of 6, and as many as 12, different genes that had variants in the BRCA1 DNA damage response pathway. Previously published case reports have shown that carriers of BRCA1 and BRCA2 mutations formed angiosarcomas following radiation treatment of breast cancer [24–26]. While these case studies did not prove causation, all three recommended consideration of BRCA status in treating primary breast carcinoma. In another study, 3 of 7 cases of radiation-induced angiosarcoma contained BRCA1 or BRCA2 mutations [27]. This group, however, concluded that BRCA status should not be considered in treatment decisions. While the percent of cases with a mutation was high, the incidence among BRCA mutation carriers was low and therefore not worth considering in treatment of the primary carcinoma. Our results may indicate the importance of the BRCA damage response outside of germline BRCA1 and BRCA2 variation. With the high number of genes affected within the tumor genome, the DNA response pathway may prove to be a promising target to combat the high rate of recurrence in these patients.

Another approach that we explored was that these tumors would have a mutational signature that is characteristic of radiation exposure. Prior work has demonstrated that non-negative matrix factorization is an analytic approach that identifies trinucleotide-based mutational signatures in tumors. This approach is capable of differentiating different mutagenic exposures, including ionizing radiation (IR) [28]. Sherborne *et al.* used this method to identify 3 mutational signatures that were characteristic of the radiation-induced tumors in a mouse model [12]. These signatures were comprised of specific patterns of tri-nucleotide variation that were not dependent upon the genetic background or the mutational load of a sample. The Beaumont angiosarcoma data alone is too limited to extract NMF signatures that can be viewed with any degree of confidence, even if the usual stability and reconstruction metrics are acceptable. To address this limitation we pooled the Beaumont angiosarcoma data with angiosarcoma data and data for known IR-induced cancers from other published studies [7–9]. The fact that this pooled full dataset analysis also yields a trinucleotide signature that is highly correlated with a previously defined IR-associated signature [9] is of interest, though this result must be viewed as tentative until confirmed based on further data for this rare cancer.

One limitation of the current study is the lack of availability of germline DNA for analysis. This hindered our ability to differentiate between somatic and germline variation. There are several variants that were identified that are often found in familial predisposition syndromes. The BRCA2 (c.1909+22delT) variant was found in 12 of the 13 radiation-associated angiosarcomas in the current study. This same variant was identified as a germline variant leading to susceptibility to breast/ovarian cancer (ClinVar: RCV000119249.1) but has recently been reannotated as benign (ClinVar:RCV000043921.6). Several others genes with potential germline variants were found when examining the “role of BRCA1 in DNA Damage Response” pathway. This included variants in ATM, BRCA1, CHEK2, FANCA, and FANCD2 that are annotated in the ClinVar database. The majority of these variants occurred in only 1 patient within our cohort. The only exception is the variant in FANCD2 (c.2613A > C / p.K871N) which is in ClinVar associated with Fanconi anemia. In addition, our study was limited by the scope of our sequencing panel. While able to detect variation in 160 genes, the panel did not allow the detection of amplification; in particular, we were unable to detect *MYC* amplification. Amplification of MYC has been shown to be an important marker of radiation-associated angiosarcoma while it is not common in sporadic angiosarcomas [29–31]. That said, *MYC* showed variants in 5 patients with other patients showing variants in genes associated with MYC mediated apoptosis signaling.

Another notable limitation is the absence of validation of the discovered variants by Sanger sequencing in order to eliminate false positives due to DNA isolation or sequencing artifacts. Unfortunately our ability for subsequent validation was eliminated by two factors in this study. First the use of laser capture microdissection greatly reduced the amount of DNA isolated from our current samples. While LCM has the benefit of focusing analysis specifically on tumor cells, the isolated DNA is only sufficient for a single sequencing run. The second important factor is the extreme rarity of this disease. As with many studies into radiation-associated angiosarcoma, we were limited by the number of available samples for study. Given the low incidence, it was difficult to compile a large cohort of available patient samples despite going back as far as 20+ years. This proved even more difficult when attempting to obtain matching recurrence and metastatic biospecimens. Ideally this study will be complemented by a future, multi-institution study with the intention of confirming and expanding this study. In order to combat these limitations, we tried to focus on variants that had evidence that indicated their potential deleterious nature. Focusing on variants categorized as pathogenic or likely pathogenic based upon ACMG guidelines, listed in HGMD/ClinVar, or having a CADD (Combined Annotation Dependent Depletion) score greater than 20 hopefully emphasized potential variants that are likely to be true positives that result in damaged phenotype.

Also of note was the absence of variants in *TP53* in the results of this study. In an earlier study of second malignant neoplasms in pediatric patients [8], identifying germline *TP53* variants was shown to be important in identifying patients at high risk for development of secondary malignancy. Of the 13 cases of radiation-associated angiosarcoma, only 4 contained variants in *TP53*. This included a case with 2 variants confirmed as somatic and pathogenic by COSMIC (COSM3388195 and COSM3378350). Interestingly, none of the 3 cases of sporadic angiosarcoma contained a TP53 variant. This suggests a role for TP53 in a subset of radiation-induced angiosarcomas and supports the conclusion of Sherborne et al that identifying germline TP53 variants prior to radiation therapy may be beneficial for treatment selection and post-treatment monitoring. However, additional studies with germline *TP53* sequencing would need to be completed on this adult population of patients with radiation-associated secondary malignancies.

Studies of radiation-induced angiosarcoma are generally limited by the low incidence, and it is important to conduct multi-center investigations to expand on the currently available studies. That being said, the current study identified individual genomic variants including variants in EGFR and BRAF that occurred in all samples of this study. In addition, genes associated with the role of BRCA1 in DNA damage response are commonly altered in all of the current cases of radiation-induced angiosarcoma. This might suggest that radiation-induced angiosarcomas are defective in the homologous recombination repair pathway which might render them sensitive to platinum-based chemotherapy and poly ADP ribose polymerase (PARP) inhibitors. Further investigation with either whole genome or exome sequencing is warranted. This would enable the discovery and confirmation of potential drug targets but also better unmask a potential radiation-associated signature.

## MATERIALS AND METHODS

### Human research protection

The Beaumont Human Investigation Committee approved all work under an approved research protocol (IRB #2014-083).

### Patient selection and laser capture microdissection (LCM)

Angiosarcoma cases from 2005 through 2015 were identified using databases available through the Department of Radiation Oncology. Inclusion criteria included previous breast cancer diagnosis and site of primary angiosarcoma to be in the ipsilateral breast as the earlier cancer. The majority of these patients received whole breast irradiation; therefore, secondary malignancies were presumed to be in-field.

Archival formalin-fixed paraffin-embedded (FFPE) tumor samples with the diagnosis of breast angiosarcoma were obtained from Beaumont Health Department of Pathology. Hematoxylin-eosin slides were reviewed by a single pathologist for confirmation of angiosarcoma and identification of tumor location. FFPE sections were cut at 5 µm thickness and mounted onto polyethylene naphthalate membrane glass slides (2 sections per slide). Before deparaffinization, slides were placed into an oven set at 60°C for 15 minutes then stained with hematoxylin and dehydrated through a series of graded ethanol and xylene steps. Pathologist-identified areas were microdissected with both UV and IR lasers using the ArcturusXT Laser Capture Microdissection system (Applied Biosystems, Carlsbad, CA) onto CapSure HS LCM Caps (Applied Biosystems, Carlsbad, CA). Four caps were filled per sample from the dissection of 1 to 4 sections.

### DNA isolation

DNA was isolated from LCM caps using GeneRead DNA FFPE Tissue Kit (Qiagen, Valencia, CA) according to manufacturer’s protocol. However, the deparaffinization step was omitted starting with the proteinase K digestion step followed by incubation at 56°C for 16 hr. Quality and amplifiable DNA material was assessed with the GeneRead DNA QuantiMIZE Kit (Qiagen, Valencia, CA).

### Library preparation

Targeted enrichment multiplex PCR of 160 genes was done using GeneRead DNAseq Comprehensive Cancer Panel V2 in combination with GeneRead DNAseq Panel PCR Kit V2 (Qiagen, Valencia, CA) following manufacturer’s protocol. Starting amount of amplifiable DNA and number of PCR cycles for initial library amplification were determined with GeneRead DNA QuantiMIZE kit. Sample purification was done using Agencourt AMPure XP Beads (Beckman Coulter, Brea, CA). Library construction was performed using GeneRead Illumina based DNA Library Prep Kits with sample multiplexing done using GeneRead Adapter I Set 12-plex (Qiagen, Valencia, CA). The resulting barcoded libraries were quantified using GeneRead DNAseq Quantification Kit, then pooled together before being submitted to a NextSeq 500 sequencer (Illumina, San Diego, CA) using a 2 × 150 paired-end sequencing strategy.

### Alignment

Primary basecall sequencing outputs were converted to FASTQ format and demultiplexed on Illumina’s BaseSpace. Read trimming and alignment were performed with NextGENe software (SoftGenetics, State College, PA). Reads with median base Phred33 quality score ≥ 10, ≤ 3 uncalled bases, ≥ 20 total bases called, and with ≤ 3 bases of quality ≤ 10 were retained for further analysis. Alignment of the sequencing reads to the human reference genome (v37.3, dbSNP 135) was performed with a NextGENe proprietary algorithm.

### Mutation analysis

Somatic mutations were called with NextGENe program on tumor only based upon mutation percentage ≥ 5% and total read coverage ≥ 250. The coverage requirement is ignored for mutations that are homozygous. If the mutation occurs with a mutation percentage ≤ 80%, the mutation is excluded if the ratio of forward and reverse reads is < 0.1. Variants were then annotated using Ingenuity Variant Analysis (IVA, 2017 Winter Release) software (Qiagen, Redwood City, CA). Variants referenced in IVA proprietary Knowledge Base with an allele frequency ≥ 1% in the general population (using the 1000 Genomes Project, the National Heart, Lung, and Blood Institute Exome Sequencing Project, the Allele Frequency Community, and the Exome Aggregation Consortium) were considered SNPs and were discarded.

### Trinucleotide-based mutational signatures

Radiation-specific alterations of genomic sequences have first been studied by applying a non-negative factorization (NMF) approach [12, 13, 28] to the filtered somatic mutations identified in our samples.

In order to extract stable signatures, the limited set of mutations detected in the angiosarcoma samples sequenced here has been complemented with previously published angiosarcoma mutation data [7] as well as with human tumor single nucleotide variants identified in malignancies known to be induced by radiation (ionizing, IR, or ultraviolet, UV) (Supplementary Table 1). The counts of the mutation motifs have then been normalized according to equal trinucleotide frequencies (following the second normalization approach used by [9, 32]. It is the second of those two normalization approaches that equalizes the probability of a purely random mutation at any one trinucleotide, which helps to isolate the variations in the data of prime interest in this work.

Thus, following previously published methods [9], we pooled normalized sequencing data for the Beaumont angiosarcoma breast cancer samples containing at least 10 mutations each (hereafter referred to as the “Bang” dataset) with normalized previously published sequencing data for the coding portion of whole genome or whole exome sequencing data for angiosarcomas [7] (the “WTSIang” dataset) as well as with normalized data for two patients whose cancers are known to have been induced by ionizing radiation (IR) [8] (the “P1-2” dataset) plus data for a third patient whose skin cancer was known to be caused by exposure to sunlight (the “P3” dataset). What is referred to below as the full dataset combines the WTSIang, the P1-2, the P3, and the Bang component datasets.

Non-negative matrix factorization (NMF) was performed following protocols developed by WTSI [13]. Signature stability and reconstruction error quality metrics were computed to estimate the number of signatures supported by the numbers of single nucleotide variants.

## SUPPLEMENTARY MATERIALS FIGURE AND TABLES




