# Supplementary material for: Mutational landscape of radiation-associated angiosarcoma of the breast
Source: Oncotarget. 2018 Jan 19;9(11):10042–53. doi: 10.18632/oncotarget.24273 (PMC5839370; doi:10.18632/oncotarget.24273)
Supplement: Supplementary file 1 [file oncotarget-09-10042-s001.pdf]

Mutational landscape of radiation-associated angiosarcoma of the breast

SUPPLEMENTARY MATERIALS

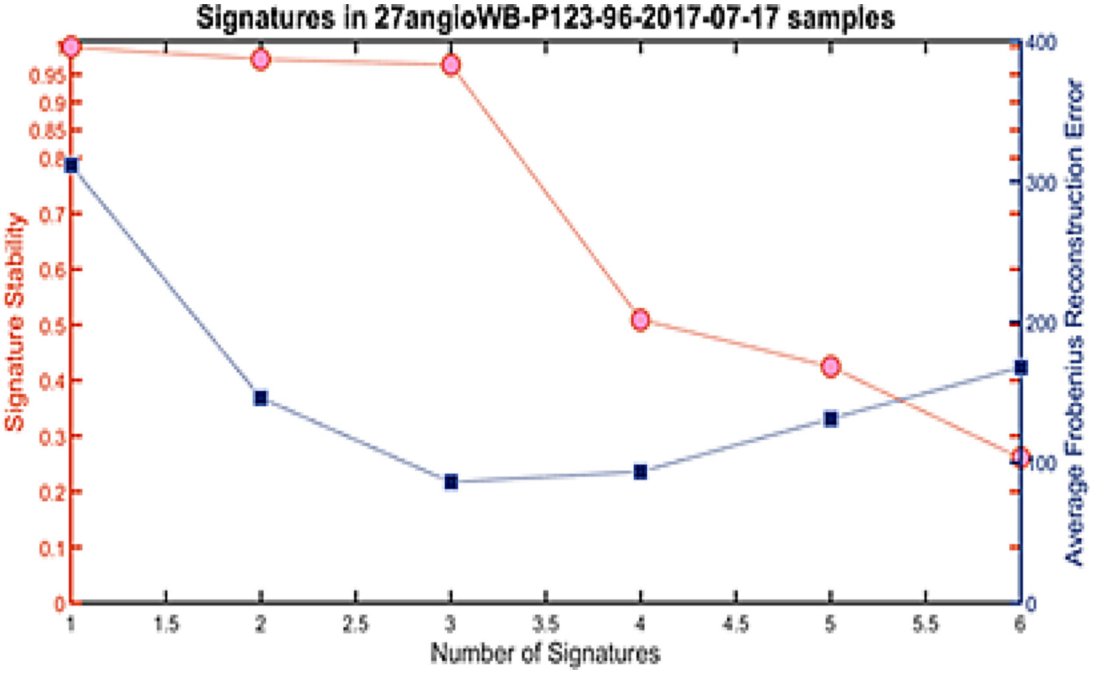

Supplementary Figure 1: Signature stability and reconstruction error for NMF applied to the Bang dataset (Beaumont angiosarcoma cases), WTSIang + P123 + Bang human-only data. Signature stability and the average Frobenius reconstruction error are shown. With the WTSIang + P123 + Bang pooled dataset 3 NMF signatures can be stably extracted.

Supplementary Table 1: Datasets used for non-negative matrix factorization (NMF) analysis

Dataset	Abbreviation	Data Type	# Samples	Institution	Ref
Beaumont Angiosarcoma	Bang	Panel of 160 genes	11	Beaumont BioBank	Beaumont BioBank
WTSI Angiosarcoma	WTSIang	Exome	11	Wellcome Trust Sanger Institute (WTSI)	[7] Behjati, S., et al., Recurrent PTPRB and PLCG1 mutations in angiosarcoma. Nat Genet, 2014. 46(4): p. 376-379.
IR-induced SMNs	P1-2	Exome	3	University of California at San Francisco (UCSF) Human	Nakamura Lab
Skin Cancer	P3	Exome	2	University of California at San Francisco (UCSF) Human	Nakamura Lab

Supplementary Table 2: Variants found in more than two-thirds of the radiation-induced angiosarcoma cases (≥ 9 of 13 cases)

Chr	Position	Gene Symbol	Ref Allele	Alt Allele	Protein Variant	Cases With Variant	Impact	Classification
1	11,293,378	MTOR	A	–	–	12	NC	VUS
1	27,100,182	ARID1A	GC	–	p.Q1327fs*10	9	FS	Likely Pathogenic
1	78,430,690	FUBP1	–	G	–	10	NC	VUS
1	120,510,722	NOTCH2	T	C	p.E414E	13	syn	Likely Benign
1	162,749,871	DDR2	CT	–	–	11	NC	VUS
1	193,111,246	CDC73	AG	–	–	11	NC	VUS
2	39,505,527	MAP4K3	A	–	–	10	NC	VUS
2	47,600,591	EPCAM	T	–	–	9	NC	VUS
2	47,600,891	EPCAM	T	–	–	13	NC	VUS
2	47,635,536	MSH2	T	–	–	10	NC	VUS
2	48,032,881	MSH6	ATCT	–	–	11	NC	VUS
2	95,843,266	ZNF2	A	T	p.T24T	9	syn	Likely Benign
2	209,101,914	IDH1	AA	–	–	12	NC	VUS
3	10,094,219	FANCD2	T	–	–	9	NC	VUS
4	55,956,186	KDR	T	C	p.K1043K	12	syn	VUS
5	56,180,645	MAP3K1	G	T	p.W1325L	12	MS	VUS
5	170,818,300	NPM1	T	–	–	13	NC	VUS
5	170,827,869	NPM1	T	A	p.N203K	12	MS	VUS
6	138,192,335	TNFAIP3	T	–	–	13	NC	VUS
7	2,966,479	CARD11	TG	–	–	9	NC	VUS
7	2,978,533	CARD11	A	T	–	9	NC	VUS
7	55,228,029	EGFR	G	A	p.C499Y	13	MS	VUS
7	140,449,164	BRAF	C	T	p.V639I	13	MS	VUS
7	140,449,185	BRAF	G	A	p.P632S	13	MS	VUS
11	108,188,279	ATM	T	–	–	12	NC	VUS
11	108,196,725	ATM	AAT	–	–	11	NC	VUS
12	56,492,754	ERBB3	TT	–	–	9	NC	VUS
13	32,907,546	BRCA2	T	–	–	12	NC	VUS
15	40,501,853	BUB1B	C	T	p.P721S	11	MS	VUS
16	2,138,213	TSC2	T	A	–	12	SSL	VUS
16	3,828,857	CREBBP	T	–	–	9	NC	VUS
16	89,874,827	FANCA	T	C	–	11	NC	VUS
17	29,528,416	NF1	T	–	–	9	NC	VUS
17	29,545,994	NF1	–	T	–	12	NC	VUS
17	41,249,370	BRCA1	A	–	–	10	NC	VUS
17	47,688,884	SPOP	–	GAGA	–	12	NC	VUS
22	41,513,744	EP300	C	T	p.G216G	10	syn	VUS
22	41,525,913	EP300	T	A	p.S396S	11	syn	Likely Benign
22	41,527,414	EP300	T	G	p.V435V	13	syn	Likely Benign
22	41,547,890	EP300	T	A	p.T957T	9	syn	VUS
22	41,565,478	EP300	T	–	–	13	NC	VUS
X	15,818,118	ZRSR2	T	–	–	10	NC	VUS
X	76,972,563	ATRX	A	–	–	13	NC	VUS
X	132,838,317	GPC3	AA	–	–	11	NC	VUS

Impact: frameshift (FS), missense (MS), synonymous (syn), splice site loss (SSL), non-coding (NC). ACMG Classification: unknown significance (VUS).

Supplementary Table 3: Variants in the pathway "Role of BRCA1 in DNA Damage Response" that were found in at least 1 case of radiation-associated angiosarcoma. See Supplementary_Table_3

Supplementary Table 4: Variants found in the matched local recurrence but not the primary radiation-induced angiosarcoma (*n* = 2 patients)

Chr	Position	Gene Symbol	Ref. Allele	Alt. Allele	Protein Variant	Impact	Classification	SIFT Function Prediction	PolyPhen-2 Function Prediction
1	65,303,705	JAK1	A	T	p.V1017E	MS	VUS	Damaging	Probably Damaging
5	1,280,340	TERT	T	G	p.D628A	MS	VUS	Damaging	Benign
5	67,589,766	PIK3R1	G	A	–	NC	VUS		
12	49,446,420	KMT2D	C	A	p.Q395H	MS	VUS		Possibly Damaging
12	56,493,721	ERBB3	C	T	p.L1013L	syn	VUS		
12	56,493,722	ERBB3	T	G	p.L1013R	MS	VUS	Tolerated	Benign
13	103,519,093	ERCC5	A	G	p.S1265G	MS	VUS		Possibly Damaging
14	95,569,740	DICER1	T	C	p.L1331L	syn	Likely Benign		
14	95,569,742	DICER1	G	A	p.L1331L	syn	Likely Benign		
14	95,570,161	DICER1	A	T	p.L1191*	SG	Pathogenic		
16	50,816,387	CYLD	A	G	–	NC	VUS		
17	37,866,649	ERBB2	C	A	p.V242V	syn	VUS		
22	41,574,596	EP300	T	A	p.L2294Q	MS	VUS	Tolerated	Possibly Damaging
X	44,733,252	KDM6A	G	A	–	NC	VUS		

Impact: missense (MS), stop gain (SG), synonymous (syn), non-coding (NC). ACMG Classification: unknown significance (VUS).

Supplementary Table 5: NMF signature correlations

	**Bang 1/2**	**Bang 2/2**	**Bang 1/3**	**Bang 2/3**	**Bang 3/3**	**WTSIang+P123+Bang 1/2**	**WTSIang+P123+Bang 2/2**	**WTSIang+P123+Bang 1/3**	**WTSIang+P123+Bang 2/3**	**WTSIang+P123+Bang 3/3**	**P123+IRmse+Uremse 1/4 (UV signature)**	**P123+IRmse+Uremse 2/4 (IRa signature)**	**P123+IRmse+Uremse 3/4 (Urethane signature)**	**P123+IRmse+Uremse 4/4 (IRb signature)**
Bang 1/2	**1.000**													
Bang 2/2	0.172	**1.000**												
Bang 1/3	**1.000**	0.170	**1.000**											
Bang 2/3	0.269	**0.978**	0.265	**1.000**										
Bang 3/3	0.193	0.600	0.196	0.460	**1.000**									
WTSIang+P123+Bang 1/2	0.438	0.444	0.440	0.464	0.265	**1.000**								
WTSIang+P123+Bang 2/2	0.249	0.817	0.245	0.853	0.330	0.207	**1.000**							
WTSIang+P123+Bang 1/3	0.389	0.447	0.391	0.463	0.260	**0.998**	0.200	**1.000**						
WTSIang+P123+Bang 2/3	0.027	**0.829**	0.024	**0.842**	0.300	0.240	**0.961**	0.246	**1.000**					
WTSIang+P123+Bang 3/3	**0.997**	0.174	**0.998**	0.265	0.218	0.418	0.248	0.369	0.020	**1.000**				
P123+IRmse+Uremse 1/4 (UV signature)	0.390	0.445	0.392	0.462	0.255	0.998	0.199	**0.999**	0.245	0.370	**1.000**			
P123+IRmse+Uremse 2/4 (IRa signature)	0.400	0.642	0.398	**0.706**	0.208	0.158	0.888	0.141	**0.790**	0.404	0.144	**1.000**		
P123+IRmse+Uremse 3/4 (Urethane signature)	-0.033	0.181	-0.037	0.181	0.102	-0.112	0.288	-0.112	0.279	-0.033	-0.114	0.238	**1.000**	
P123+IRmse+Uremse 4/4 (IRb signature)	-0.063	0.320	-0.067	0.336	0.043	-0.022	0.484	-0.019	0.508	-0.069	-0.020	0.369	0.027	**1.000**
