# Supplementary material for: Mutational landscape of radiation-associated angiosarcoma of the breast
Source: Oncotarget. 2018 Jan 19;9(11):10042–53. doi: 10.18632/oncotarget.24273 (PMC5839370; doi:10.18632/oncotarget.24273)
Supplement: Supplementary file 2 [file oncotarget-09-10042-s002.docx]

**Supplementary Table 3 : Variants in the pathway “Role of BRCA1 in DNA Damage Response” that were found in at least 1 case of radiation-associated angiosarcoma.** Impact: frameshift (FS), missense (MS), stop gain (SG), synonymous (syn), splice site loss (SSL). ACMG Classification: unknown significance (VUS).

| **Chr** | **Position** | **Gene Symbol** | **Ref. Allele** | **Alt. Allele** | **Protein Variant** | **Case With Variant** | **Impact** | **Classification** | **SIFT Function Prediction** | **PolyPhen-2 Function Prediction** |
| --- | --- | --- | --- | --- | --- | --- | --- | --- | --- | --- |
| 1 | 27,094,320 | ARID1A | A | T | p.K1010* | 1 | SG | Pathogenic |  |  |
| 1 | 27,100,166 | ARID1A | C | A | p.P1321H | 1 | MS | VUS | Damaging | Probably Damaging |
| 1 | 27,100,182 | ARID1A | GC | – | p.Q1327fs*10 | 9 | FS | Likely Pathogenic |  |  |
| 1 | 27,100,305 | ARID1A | T | A | p.Y1339* | 1 | SG | Pathogenic |  |  |
| 1 | 27,105,577 | ARID1A | A | T | p.I1730F | 1 | MS | VUS | Damaging | Possibly Damaging |
| 1 | 27,105,643 | ARID1A | A | T | p.K1752* | 1 | SG | Pathogenic |  |  |
| 1 | 27,105,842 | ARID1A | A | T | p.Q1818L | 1 | MS | VUS | Damaging | Benign |
| 1 | 27,107,212 | ARID1A | A | T | p.I2058F | 1 | MS | VUS | Damaging | Probably Damaging |
| 2 | 47,656,925 | MSH2 | A | T | p.Q374L | 1 | MS | VUS | Damaging | Benign |
| 2 | 47,703,668 | MSH2 | C | T | p.S657F | 1 | MS | VUS | Damaging | Probably Damaging |
| 2 | 47,707,926 | MSH2 | A | T | p.E850D | 1 | MS | VUS | Damaging | Benign |
| 2 | 48,026,404 | MSH6 | A | T | p.K126* | 1 | SG | Pathogenic |  |  |
| 2 | 48,026,677 | MSH6 | A | T | p.K519* | 1 | SG | Pathogenic |  |  |
| 2 | 48,027,638 | MSH6 | A | G | p.D709G | 2 | MS | VUS | Damaging | Probably Damaging |
| 2 | 48,027,639 | MSH6 | – | T | p.S710* | 2 | FS | Likely Pathogenic |  |  |
| 3 | 10,076,481 | FANCD2 | A | T | p.S126C | 1 | MS | VUS | Damaging | Probably Damaging |
| 3 | 10,089,625 | FANCD2 | A | T | p.I435F | 1 | MS | VUS | Damaging | Possibly Damaging |
| 3 | 10,089,628 | FANCD2 | C | A | p.L436M | 1 | MS | VUS | Damaging | Probably Damaging |
| 3 | 10,089,664 | FANCD2 | C | T | p.Q448* | 1 | SG | Pathogenic |  |  |
| 3 | 10,089,671 | FANCD2 | T | G | p.I450R | 1 | MS | VUS | Damaging | Possibly Damaging |
| 3 | 10,114,944 | FANCD2 | A | C | p.K871N | 3 | MS | VUS | Damaging | Benign |
| 3 | 10,115,018 | FANCD2 | C | T | p.T896M | 1 | MS | VUS | Damaging | Possibly Damaging |
| 3 | 10,133,929 | FANCD2 | T | A | p.L1281* | 1 | SG | Pathogenic |  |  |
| 3 | 10,134,984 | FANCD2 | C | T | p.P1289S | 1 | MS | VUS | Damaging | Possibly Damaging |
| 3 | 10,138,093 | FANCD2 | G | A | p.M1374I | 1 | MS | VUS | Damaging | Probably Damaging |
| 3 | 52,643,681 | PBRM1 | T | A | p.N739Y | 1 | MS | VUS | Damaging | Probably Damaging |
| 3 | 52,651,469 | PBRM1 | A | T | p.C543S | 1 | MS | VUS | Tolerated | Probably Damaging |
| 3 | 52,668,729 | PBRM1 | C | T | p.R397Q | 1 | MS | VUS | Tolerated | Probably Damaging |
| 3 | 52,676,024 | PBRM1 | T | A | p.I345F | 1 | MS | VUS | Damaging | Possibly Damaging |
| 3 | 52,712,543 | PBRM1 | T | C | p.E70G | 1 | MS | VUS | Damaging | Probably Damaging |
| 6 | 35,430,660 | FANCE | T | C | p.M493T | 7 | MS | VUS |  | Possibly Damaging |
| 11 | 108,114,784 | ATM | C | A | p.Q201K | 1 | MS | VUS | Damaging | Probably Damaging |
| 11 | 108,114,788 | ATM | C | T | p.T202I | 1 | MS | VUS | Damaging | Probably Damaging |
| 11 | 108,119,823 | ATM | T | C | p.V410A | 1 | MS | Benign | Damaging | Benign |
| 11 | 108,124,630 | ATM | T | G | p.L663* | 1 | SG | Pathogenic |  |  |
| 11 | 108,126,940 | ATM | A | T | – | 1 | SSL | Pathogenic |  |  |
| 11 | 108,139,202 | ATM | A | T | p.K902* | 1 | SG | Pathogenic |  |  |
| 11 | 108,175,571 | ATM | T | A | p.L1889* | 1 | SG | Pathogenic |  |  |
| 11 | 108,178,706 | ATM | A | T | p.Q1919H | 3 | MS | VUS | Damaging | Probably Damaging |
| 12 | 46,205,329 | ARID2 | T | A | p.V138E | 1 | MS | VUS |  | Possibly Damaging |
| 12 | 46,244,191 | ARID2 | T | A | p.L762* | 1 | SG | Pathogenic |  |  |
| 12 | 46,244,923 | ARID2 | T | A | p.M1006K | 4 | MS | VUS | Damaging | Possibly Damaging |
| 12 | 46,244,932 | ARID2 | T | A | p.V1009E | 4 | MS | VUS | Tolerated | Possibly Damaging |
| 12 | 46,245,349 | ARID2 | T | A | p.L1148* | 1 | SG | Pathogenic |  |  |
| 12 | 46,245,384 | ARID2 | C | T | p.P1160S | 1 | MS | VUS | Tolerated | Possibly Damaging |
| 13 | 32,890,613 | BRCA2 | A | T | p.K6* | 1 | SG | Pathogenic |  |  |
| 13 | 32,906,872 | BRCA2 | T | A | p.C419* | 2 | SG | Pathogenic |  |  |
| 13 | 32,911,211 | BRCA2 | A | T | p.K907* | 1 | SG | Pathogenic |  |  |
| 13 | 32,911,422 | BRCA2 | T | A | p.L977* | 1 | SG | Pathogenic |  |  |
| 13 | 32,914,281 | BRCA2 | T | G | p.L1930* | 6 | SG | Pathogenic |  |  |
| 13 | 32,929,177 | BRCA2 | T | A | p.L2396* | 2 | SG | Pathogenic |  |  |
| 13 | 32,945,132 | BRCA2 | A | T | p.N2843Y | 2 | MS | VUS |  | Probably Damaging |
| 13 | 48,953,752 | RB1 | T | A | p.L452* | 1 | SG | Pathogenic |  |  |
| 13 | 49,039,437 | RB1 | C | T | p.P808S | 1 | MS | VUS | Damaging | Probably Damaging |
| 16 | 89,813,062 | FANCA | G | A | p.P1148L | 1 | MS | VUS | Damaging | Probably Damaging |
| 16 | 89,813,063 | FANCA | GG | – | p.P1148fs*66 | 1 | FS | Likely Pathogenic |  |  |
| 16 | 89,813,064 | FANCA | G | – | p.S1149fs*2 | 1 | FS | Likely Pathogenic |  |  |
| 16 | 89,818,547 | FANCA | T | A | p.Q1022L | 3 | MS | VUS | Damaging | Probably Damaging |
| 16 | 89,828,388 | FANCA | G | A | p.P941S | 1 | MS | VUS | Tolerated | Possibly Damaging |
| 16 | 89,849,443 | FANCA | A | T | p.L513* | 1 | SG | Pathogenic |  |  |
| 16 | 89,849,488 | FANCA | A | T | p.L498Q | 2 | MS | VUS | Damaging | Probably Damaging |
| 16 | 89,858,433 | FANCA | T | A | p.Q376L | 7 | MS | VUS | Damaging | Benign |
| 16 | 89,874,730 | FANCA | T | A | p.I190F | 3 | MS | VUS | Damaging | Benign |
| 16 | 89,880,985 | FANCA | T | A | p.K76* | 1 | SG | Pathogenic |  |  |
| 17 | 7,578,203 | TP53 | C | T | p.V216M | 1 | MS | Pathogenic | Damaging | Probably Damaging |
| 17 | 7,578,212 | TP53 | G | A | p.R81* | 1 | SG | Pathogenic |  |  |
| 17 | 41,243,634 | BRCA1 | T | A | p.D1258V | 1 | MS | VUS | Damaging | Possibly Damaging |
| 17 | 41,246,406 | BRCA1 | T | A | p.K381I | 2 | MS | VUS | Damaging | Probably Damaging |
| 17 | 41,256,944 | BRCA1 | T | A | p.Q81L | 1 | MS | VUS | Damaging | Possibly Damaging |
| 17 | 41,256,960 | BRCA1 | T | A | p.S76C | 1 | MS | VUS | Damaging | Probably Damaging |
| 17 | 41,256,961 | BRCA1 | T | A | p.E75D | 1 | MS | VUS | Tolerated | Possibly Damaging |
| 17 | 59,853,886 | BRIP1 | C | T | p.R658Q | 1 | MS | VUS | Tolerated | Probably Damaging |
| 17 | 59,876,545 | BRIP1 | C | T | p.R419Q | 1 | MS | VUS | Tolerated | Probably Damaging |
| 19 | 11,096,925 | SMARCA4 | C | T | p.P139L | 1 | MS | VUS | Tolerated | Probably Damaging |
| 19 | 11,141,552 | SMARCA4 | G | A | p.D1177N | 1 | MS | VUS | Damaging | Probably Damaging |
| 19 | 11,144,174 | SMARCA4 | A | G | p.E1252G | 1 | MS | VUS | Damaging | Benign |
| 19 | 11,172,463 | SMARCA4 | C | T | p.R1608C | 1 | MS | VUS | Damaging | Benign |
| 19 | 11,172,466 | SMARCA4 | T | C | p.S1606P | 1 | MS | VUS | Tolerated | Possibly Damaging |
| 22 | 24,143,196 | SMARCB1 | A | C | p.D143A | 1 | MS | VUS | Damaging | Probably Damaging |
| 22 | 24,167,427 | SMARCB1 | G | A | p.G262R | 1 | MS | VUS | Damaging | Probably Damaging |
| 22 | 24,175,770 | SMARCB1 | T | A | p.L324Q | 2 | MS | VUS | Damaging | Probably Damaging |
| 22 | 24,175,793 | SMARCB1 | C | T | p.R332W | 1 | MS | VUS | Damaging | Probably Damaging |
| 22 | 29,091,740 | CHEK2 | C | T | p.R377H | 1 | MS | VUS | Tolerated | Possibly Damaging |
| 22 | 29,121,087 | CHEK2 | A | G | p.I200T | 1 | MS | VUS | Tolerated | Possibly Damaging |
